# Preventing Road Deaths—Time for Data

**DOI:** 10.1371/journal.pmed.1000257

**Published:** 2010-03-30

**Authors:** The PLoS Medicine Editors

## Abstract

In this month's editorial, the *PLoS Medicine* Editors call for better data to support policy changes that could reduce the huge global burden of death and injury from road traffic crashes.

This week in *PLoS Medicine* we publish a Policy Forum article by Lagarde and Constant on the prevention of injuries and deaths in vulnerable road users (VRUs) [1] and a research article by Shankuan Zhu and colleagues examining whether body mass index (BMI) is a risk factor for injuries sustained in road traffic crashes [2]. Following the recent refocusing of the journal's scope [3] we are now keen to prioritize studies that examine all factors—whether environmental, economic, political, or biological—that contribute substantially to morbidity and mortality worldwide. This includes studies of road injuries and deaths.

A recent WHO report that highlighted the public health problems associated with inadequate road safety opened with some stark figures: 1.2 million deaths and up to 50 million nonfatal injuries occur every year on the world's roads [4]. And it seems these numbers are set to increase. In 2006, Mathers and Loncar predicted a 40% increase in deaths due to injury between 2002 and 2030, and that the majority of these deaths would be due to increasing numbers of road traffic crashes [5]. Economic growth around the world, especially in low- and middle-income countries, should bring with it health benefits, but the authors cautioned that increased wealth means more cars on the roads, which would likely result in a concomitant increase in road injuries and deaths [5]. Reducing the toll of these road deaths can be achieved only by a combination of environmental factors (such as build quality of roads, incorporation of pedestrian and cycle paths) improved vehicle and road safety (through appropriate legislation on vehicle safety, vehicle speed, and factors such as alcohol and other drug use by drivers), and promotion and improvement of safe alternatives to driving.

Although it is vital to ensure that drivers and passengers in vehicles are protected from potential injury, we must not forget that VRUs—pedestrians, cyclists, motorcyclists—account for almost half of those who die on roads [4]. Indeed, far less attention has been focused on reducing deaths among those not travelling in cars than on safety of car users. As Lagarde and Constant point out, that there are “clear deficiencies in data collection,” meaning that we don't really have an accurate picture of how many cyclists and pedestrians are dying or injured on roads around the world. In many countries a substantial number of road crashes are not even reported to the police, or, if police are involved, there seems to be no obligation to record injuries to VRUs [1]. The level of under-reporting varies considerably from country to country, but studies have consistently demonstrated gross under-reporting of particularly nonfatal road traffic injuries. For instance, in a cross-sectional survey of more than 10,000 people carried out in urban India, even fatal road traffic injuries were under-reported, with 22% of fatalities not reported to the police [6]. We need local evidence as well as global estimates, because local studies are required by policymakers to allocate resources and put in place appropriate interventions to tackle this problem.

The importance of obtaining and acting on a robust, detailed evidence base for injury and deaths cannot be overstated. According to Patton et al. [7], traffic crashes are the single greatest cause of mortality among young people (aged 10–24 y) worldwide. How much do we know about the range and types of injuries sustained in road crashes and the risk factors involved? There may be specific risk factors for people of different ages and cultural groups, for men and for women. For example, self-harm was found to be an independent risk factor for motor vehicle crashes in young drivers, in a recent cohort-based study [8]. To what extent do speed reductions, educational tools, reduction of traffic volumes, and promotion of alternatives to vehicular transport avert injuries and deaths on the roads, especially in resource-limited settings? This is also a ripe area for multidisciplinary research: for example, innovative analyses can reveal where pedestrian injury and death hotspots are and link them to the quality of the built environment, enabling city planners to focus on those areas where countermeasures needs to be put in place in a systematic fashion to improve safety for all pedestrians [9]. Clearly, medical journals have a duty to publish studies that examine the full range of road traffic crash mitigation measures, including legislation, use of protective clothing, road build quality, and even lighting, if we are to build an evidence base for effective policymaking.

Global initiatives to tackle road safety are already in place. The Global Road Safety Initiative (GRSI) (http://www.grsproadsafety.org/page-grsi-42.html), which is funded by seven of the world's largest car and oil companies, aims to implement the World Report on Road Traffic Injury Prevention (WHO and World Bank, 2004) [10]. The GSRI initiative ran from 2005 to 2009 and was put in place by the Global Road Safety Partnership (http://www.grsproadsafety.org/). The funds were targeted at road safety improvement in China and Brazil. An interim report [11] shows that US$10 billion were spent on measures such as education on wearing helmets and on drinking and driving, and improvements in safety at road junctions, better signage, and setting up local crash databases to improve local evidence bases. Critical and independent evaluation of this initiative should provide valuable information for policymakers aiming to implement similar measures in their own countries. Another global initiative includes a resolution on Global Road Safety, which was introduced during the 62nd Session of the UN General Assembly on 31 March 2008. The outcomes of this meeting were articulated clearly by the chair of the commission Lord George Robertson: “This resolution can mark the moment when the world's community looks out at the suffering and the grief and the cost of road crashes and decides to end it. This is in our power to do. We have the tools, we have the knowledge and we have the means. What we have to do now is act” [12].

A prerequisite for ameliorating injuries and preventing deaths is good-quality data that is widely disseminated; this is where we believe *PLoS Medicine* has a role to play. Research into the risk factors for injury from road traffic crashes, analyses of attitudes to road safety, evaluation of projects aiming to improve road safety, and alternatives to motor vehicle travel are all areas where medical researchers, statisticians, and public health professionals can contribute vital information in support of life-saving policies. Data are needed from both rich and poor settings, as we should remember that 91% of road deaths occur in low- and middle-income countries. We look forward to publishing more such studies from a variety of disciplines and settings in the coming years.

## Abbreviations

BMI: body mass index
GRSI: Global Road Safety Initiative
VRU: vulnerable road user
